# Expression of DOCK9 and DOCK11 Analyzed with Commercial Antibodies: Focus on Regulation of Mutually Exclusive First Exon Isoforms

**DOI:** 10.3390/antib9030027

**Published:** 2020-06-27

**Authors:** Antonio Parrado

**Affiliations:** Instituto Murciano de Investigación Biosanitaria Virgen de la Arrixaca (IMIB-Arrixaca), 30120 Murcia, Spain; antonio.parrado@carm.es

**Keywords:** DOCK9, DOCK11, Zizimin, expression, alternative splicing

## Abstract

Dedicators of cytokinesis 9 and 11 (DOCK9 and DOCK11) are members of the dedicator of cytokinesis protein family encoding the guanosine nucleotide exchange factors for Rho GTPases. Together with DOCK10, they constitute the DOCK-D or Zizimin subfamily. Two alternative full-length amino terminal isoforms of DOCK9 are known, which we will call DOCK9.1 and DOCK9.2. In order to investigate the relevance of the presence of the alternative first exon isoforms within this family, and to lay the groundwork for future studies that seek to investigate their potential role as biomarkers of disease, the expression levels of DOCK9 and DOCK11 were measured by qRT-PCR in 26 human tissues and 23 human cell lines, and by Western blot analysis, using commercial antibodies in cell lines. DOCK9.1 and DOCK9.2 were widely distributed. High levels of expression of both isoforms were found in the lungs, placenta, uterus, and thyroid gland. However, only DOCK9.1 was significantly expressed in the neural and hematopoietic tissues. The unique first exon form of DOCK11 was highly expressed in hematopoietic tissues, such as the peripheral blood leukocytes, spleen, thymus, or bone marrow, and in others such as the lungs, placenta, uterus, or thyroid gland. In contrast to tissues, the expression of DOCK9.1 and DOCK9.2 differed from one another and also from total DOCK9 in cell lines, suggesting that the amino terminal isoforms of DOCK9 may be differentially regulated. This study demonstrates the usefulness of antibodies in investigating the regulation of the expression of DOCK9.1, total DOCK9, and DOCK11.

## 1. Introduction

Dedicator of cytokinesis (DOCK) is the designation for a family of 11 genes that encode the large guanosine nucleotide exchange factors (GEF) for Rho GTPases. DOCK proteins are characterized by a GEF domain called CZH2 [1,2,3]. They play roles in cell shape and movement by regulating actin cytoskeleton dynamics. The DOCK-D or Zizimin subfamily consists of three members, DOCK9, DOCK10, and DOCK11. DOCK9 and DOCK11 share a higher structural homology with each other than with DOCK10. Consistent with their preference for GTPase Cdc42, DOCK9 and DOCK11 promote the formation of filopodia [4,5,6,7,8].

At the mRNA level, DOCK9 is highly expressed in the placenta, lungs, brain, kidneys and thyroid, and DOCK11 is expressed in the peripheral blood leukocytes, thymus, spleen, placenta, lungs, kidneys, and thyroid [5,9]. DOCK9 has two alternative full-length amino terminal isoforms, designated here as DOCK9.1 and DOCK9.2 by analogy with DOCK10 (NCBI RefSeq database numbers NM_015296 and NM_001130048, respectively) [10,11,12,13]. Only one full-length amino terminal form of DOCK11 has been described (RefSeq no. NM_144658). In terms of their size, the mutually exclusive first exons represent only ~2% of the total size of the DOCK9 isoforms, and lack recognizable functional domains [14,15,16,17], which suggests that the isoforms, rather than functional diversity, may provide alternative mechanisms for the control of DOCK9 expression. The expression of the DOCK9 isoforms has not been studied thus far.

In accordance with their function in cell plasticity, DOCK9 and DOCK11 could play roles in cancer and other pathologies. The abnormal expression of DOCK9 has been found in glioblastoma [18], papillary thyroid carcinoma [19], tuberculosis [20], prostate cancer [21], and pancreatic cancer [22], and of DOCK11 in testicular carcinoma [23]. The implementation of methods for studying the expression of DOCK9 and DOCK11 may help validate these alterations in large numbers of patients.

In this paper, the mRNA levels of DOCK9.1 and DOCK9.2, total DOCK9 and total DOCK11 were measured in human primary tissues, and the mRNA and protein levels in human cell lines were assessed using commercial antibodies (Abs). The tissue distribution of DOCK9.1 and DOCK9.2 was similar, but in cell lines DOCK9.2 had a more restricted expression, suggesting that the isoforms undergo differential and post-transcriptional regulation. The data also suggested that there are no alternative first exon isoforms of DOCK11. These findings may be useful for future studies aimed at establishing the potential roles of DOCK9 and DOCK11 as biomarkers of disease.

## 2. Materials and Methods

### 2.1. Samples

A collection of RNA samples from pooled human tissues was purchased from Clontech (Takara Bio USA, Mountain View, CA, USA). The panel included 26 different tissues: brain, cerebellum, fetal brain, spinal cord, kidney, liver, trachea, heart, skeletal muscle, uterus, colon, small intestine, stomach, placenta, prostate, testis, adrenal gland, salivary gland, thyroid gland, fetal liver, spleen, thymus, tonsil, leukocytes and bone marrow.

A panel of 23 human hematopoietic and epithelial cell lines, detailed in Supplementary Table S1, was used. Hematopoietic cells were grown in RPMI-1640 medium supplemented with 10% fetal calf serum (Biowhittaker, Cambrex, East Rutherford, NJ, USA), 50 U/mL penicillin, 50 U/mL streptomycin, 2.5 μg/mL amphotericin B, and 2 mM l-glutamine. Epithelial cells were grown in Dulbecco’s minimum essential medium (DMEM) with the same supplements.

### 2.2. Transient Transfections

Human embryonic kidney 293T cells were cultured in DMEM with the same supplements, transfected at subconfluency with plasmid constructs for the transient expression of DOCK9.1, DOCK10.1 and DOCK11 (Supplementary Materials, Table S2) using lipofectamine reagent, and were cultured for 24 h.

### 2.3. qRT-PCR

Total RNA was obtained with the SV Total RNA Isolation Kit (Promega, Madison, WI, USA). One μg of RNA was retrotranscribed using the High-Capacity cDNA Reverse Transcription Kit (Applied Biosystems, Waltham, MA, USA), followed by PCR in an ABI Prism 7000 Sequence Detection System. Details of the Taqman assays used are shown in Supplementary Table S3. These included two predesigned assays to detect total human DOCK9, one directed to the boundaries between exons 27 and 28, and the other to the boundary between exons 33 and 34. Two predesigned assays, directed to the boundaries between the respective exon 1 of DOCK9.1 and DOCK9.2 and the common exon 2 were used to detect the alternative first exon splicing isoforms of DOCK9. Two assays were used to detect human DOCK11: one custom assay, directed to the boundary between exons 1 and 2 and another predesigned assay, directed to the boundary between exons 36 and 37. Lastly, a predesigned GAPDH assay was performed for normalization. All measurements were performed in duplicate. Relative expression levels were calculated by the ΔΔCt method [24].

### 2.4. Western Blot Analysis

Twenty µg of total protein extracted from the cell lines was electrophoresed in 6% SDS-PAGE gels, electroblotted onto nitrocellulose filters, and incubated with rabbit polyclonal Abs from Bethyl Laboratories (Montgomery, TX, USA) against DOCK9 (ref. nos. 530A, 531A, and 532A, mapping to amino acids 1–50, 1250–1300, and 1850–1900, respectively), DOCK10 (ref. no. 305A), and DOCK11 (ref. nos. 638A and 639A, mapping to amino acids 100–150 and 400–450, respectively), followed by swine anti-rabbit Igs, HRP. Duplicate blots, obtained after electrophoresis of the protein extracts in 10% gels, were probed with GAPDH Ab, HRP, as a normalization control. Details about the use of the Abs are shown in Supplementary Table S4. Blots were developed with ECL reagent, and quantitated in a ChemiDoc XRS+ photodocumentation system using the Image Lab software (Bio-Rad Laboratories, Hercules, CA, USA).

### 2.5. Statistical Analysis

The correlation between assays was evaluated by linear regression analysis. Statistical significance was established for *p* < 0.05. Pearson correlation coefficient (R) values close to one indicate high association. 

## 3. Results

### 3.1. Expression of Mutually Exclusive First Exon Isoforms of DOCK9 in Human Tissues

**Figure 1:**
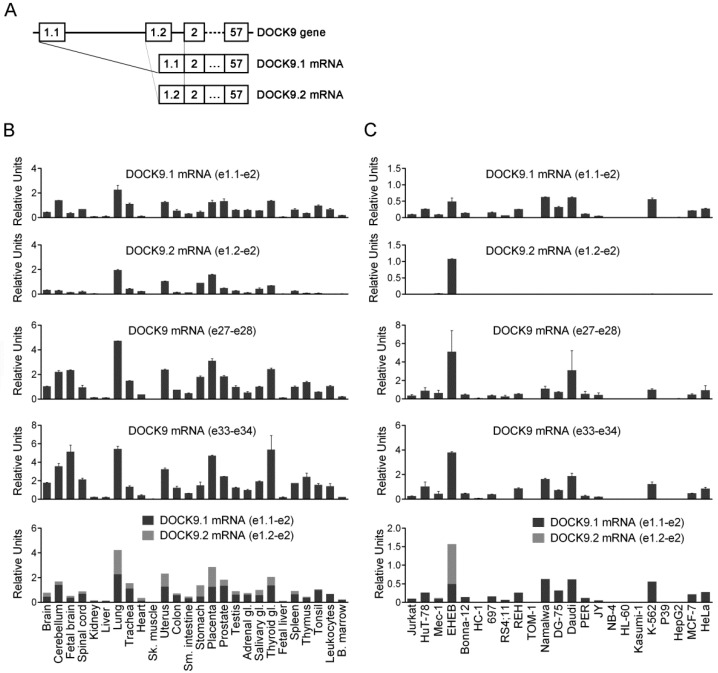
mRNA expression of dedicator of cytokinesis 9 (DOCK9) and its alternative first exon isoforms in human tissues and cell lines. (**A**) Schematic representation of the DOCK9 gene and the DOCK9 isoforms indicating the exons that constitute them. (**B**) qRT-PCR analysis of DOCK9.1, DOCK9.2, and total DOCK9 using two different assays in 26 human tissues. The bottom chart represents the overlap of the mRNA levels of DOCK9.1 and DOCK9.2, and is shown to demonstrate their equivalence to total DOCK9. (**C**) qRT-PCR analysis of DOCK9.1, DOCK9.2, and total DOCK9 in 23 human cell lines, displayed as in (**B**). All experiments were performed in duplicate (mean ± S.D.).

A schematic representation of the two alternative amino terminal full length isoforms of DOCK9 is shown in Figure 1A. Specific qRT-PCR assays were used to analyze the expression of DOCK9.1, DOCK9.2 and total DOCK9 in a panel of 26 RNA samples from human tissues (Figure 1B and Supplementary Table S5). For total DOCK9, two assays located in different regions were used, in order to minimize potential biases produced by alternative splicing. The expression of DOCK9.1 and DOCK9.2 significantly correlated in human tissues (Table 1), indicating that both isoforms exhibited a roughly similar distribution, which is evident in tissues with a high expression of DOCK9 such as the lungs, placenta and uterus. However, significant differences were found in diverse tissues, such as neural tissues (e.g., cerebellum, spinal cord) and hematopoietic tissues (e.g., spleen, thymus, tonsils, leukocytes), which showed higher levels of DOCK9.1 than DOCK9.2. Both total DOCK9 assays highly correlated with each other, though DOCK9 e27-e28 was better associated with the isoform-specific assays. The best correspondence was found between the DOCK9 e27-e28 assay and the sum of the isoform-specific assays.

### 3.2. Expression of Mutually Exclusive First Exon Isoforms of DOCK9 in Human Cell Lines

DOCK9 mRNA expression was also studied in a panel of 23 human cell lines, which included B-lymphoid cell lines, but also myeloid, T-lymphoid, and nonhematopoietic cell lines (Figure 1C and Supplementary Table S5). DOCK9.1 and total DOCK9 were widely distributed, and high expression levels were displayed by several B cell lines (EHEB, Namalwa, Daudi, DG-75, RS4; 11), T cell line HuT-78, myeloid cell line K-562, and epithelial cell line HeLa. DOCK9.2 mRNA was found to be highly expressed in EHEB and was also detected, at much lower levels, in another B cell line, Mec-1. Since most of the cell lines used in this study are hematopoietic, the absence of DOCK9.2 expression in cell lines is consistent with the lack of DOCK9.2 expression in hematopoietic tissue (Figure 1B). Therefore, it was not surprising that the expression of DOCK9.1 and DOCK9.2 did not correlate in cell lines (Table 1). However, both total DOCK9 assays correlated with each other and with the isoform-specific assays. The best association in cell lines was found between DOCK9 e33-e34 and the sum of the isoform-specific assays.

### 3.3. Protein Expression of DOCK9 in Human Cell Lines

**Figure 2:**
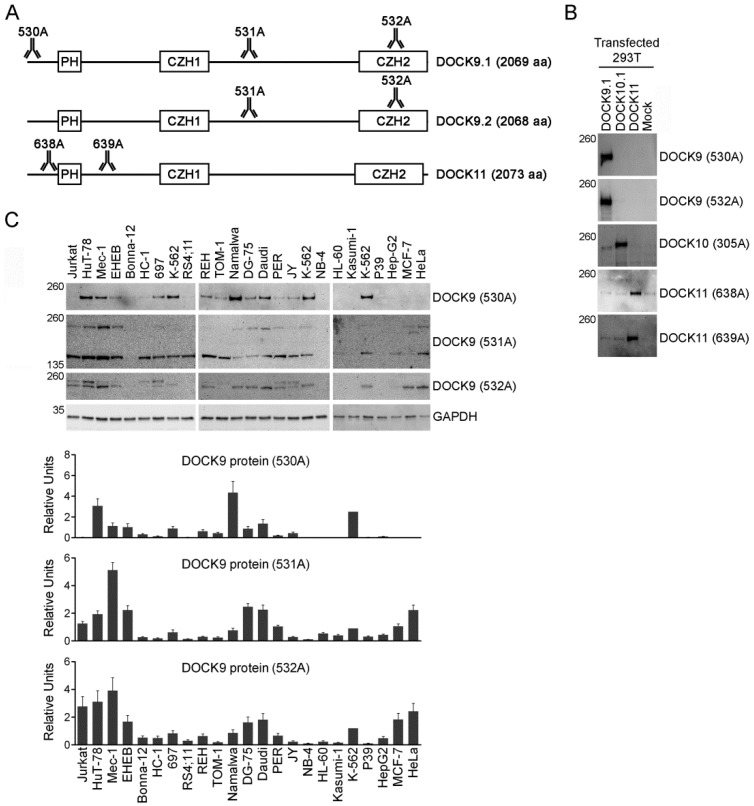
Expression of DOCK9 protein in human cell lines. (**A**) Schematic representation of the DOCK9 isoforms, and of the DOCK11 protein, indicating the approximate positions targeted by the antibodies (Abs) used in the present study. (**B**) Western blot analysis of 293T cells transfected with expression vectors for the indicated DOCK-D family members, using Abs directed to DOCK9, DOCK10 or DOCK11. (**C**) Western blot analysis of 23 human cell lines, using Abs directed to DOCK9 and GAPDH. The positions of the size markers are indicated in kDa to the left. References of the Abs are shown to the right. Charts represent quantitation of two replicate blots (mean ± S.D.). Only the 236 kDa band has been quantified.

A schematic representation of DOCK9 isoforms and DOCK11 is depicted in Figure 2A, showing the approximate regions targeted by the Abs. DOCK9 Abs 530A and 532A and DOCK11 Abs 638A and 639A were specific, as demonstrated by the detection of a band at their expected size of 236 kDa and 237 kDa, respectively, by Western blot analysis of 293T cells transfected with DOCK9.1, DOCK10.1 and DOCK11, in the appropriate lanes (Figure 2B).

The human cell lines were analyzed by Western blot using the DOCK9 Abs. Quantitation was normalized using the DOCK9 signal in the K-562 cell line, which was included in all the blots as a reference. The 530A Ab recognizes DOCK9.1, which showed its highest levels in the B cell line Namalwa, T cell line HuT-78 and myeloid cell line K-562 (Figure 2B). The 532A Ab recognizes a common C-terminal region of DOCK9, which showed its highest levels in B cell lines Mec-1, Daudi, DG-75 and EHEB, T cell lines HuT-78 and Jurkat, myeloid cell line K-562, and epithelial cell lines HeLa and MCF-7. The results using 530A and 532A disagreed (Table 2). The presence of an additional band of approximately 250 kDa, possibly due to cross reaction with DOCK10, prompted to use the 531A Ab, which recognizes a common central region of DOCK9, despite the fact that the package insert discourages its use in immunoblotting. The 531A Ab showed expression of a band at the expected size of 236 kDa and another of smaller size, with similar profile to the former but higher expression. This smaller band could correspond to isoforms with truncated carboxy terminal ends (NP_001123521, NP_001123522) [10] which, according to the ProtParam tool [25], have an expected size of 142 kDa. The levels of full length DOCK9 detected with 531A were similar as those detected with 532A, and their results agreed. The results of 530A and 531A were not related.

The expression of DOCK9.1 mRNA was significantly associated to the expression of DOCK9 protein analyzed with 530A, but not with 531A or 532A, in cell lines. Significant correlations, but with lower p values, were also found between DOCK9 mRNA and protein using DOCK9 e33-e34 and 530A, and DOCK9 e27-e28 and 531A, and in no other comparison, suggesting a relatively poor correspondence between total DOCK9 mRNA and protein expressions. A representative example of this discrepancy is provided by the B cell line EHEB, which showed high mRNA levels, but much lower protein levels.

### 3.4. Expression of DOCK11 mRNA in Human Tissues and Cell Lines

**Figure 3:**
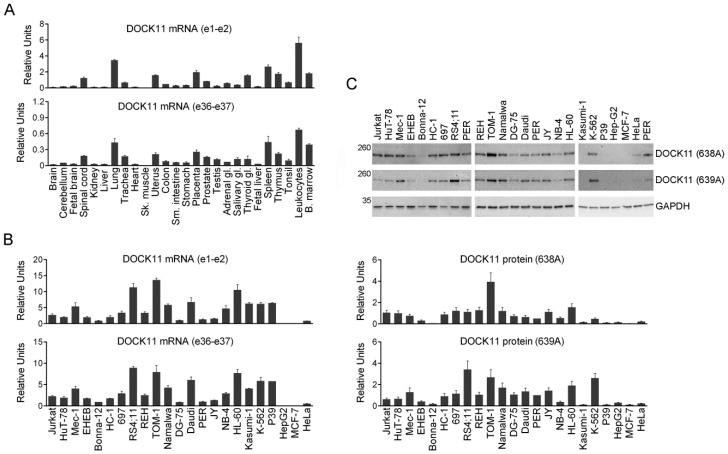
Expression of DOCK11 mRNA and protein in human tissues and cell lines. (**A**) qRT-PCR analysis of DOCK11 using two different assays in 26 human tissues. (**B**) qRT-PCR analysis of DOCK11 in 23 human cell lines. All experiments were performed in duplicate (mean ± S.D.). (**C**) Western blot analysis of 23 human cell lines, using DOCK11 Abs (depicted in Figure 2A) and a GAPDH Ab. The positions of the size markers are indicated in kDa to the left. References of the Abs used are shown to the right. Charts represent quantitation of two replicate blots (mean ± S.D.).

To search for evidence of potential amino terminal forms of DOCK11, in addition to the one already described, two qRT-PCR assays have been used, one directed to the boundary between exons 1 and 2 and the other to the central region, in the same panels of tissues (Figure 3A) and cell lines (Figure 3B). DOCK11 e1-e2 showed higher sensitivity than DOCK11 e36-e37 as suggested by its lower ∆Ct values (Supplementary Table S5). The mRNA levels measured using both assays coincided, both in human tissues and cell lines (Table 1), suggesting that the known amino terminal form of DOCK11 is the only one that exists.

### 3.5. Protein Expression of DOCK11 in Human Cell Lines

The cell lines were tested for protein expression using the DOCK11 Abs. Quantitation was normalized using the DOCK11 signal in the PER cell line, which was included in all the blots as a reference. The 638A and 639A Abs recognized common regions of DOCK11, and they displayed relatively similar results between each other (Figure 3C) and with mRNA levels (Table 3), suggesting a good correspondence between DOCK11 mRNA and protein expressions.

## 4. Discussion

In previous studies, DOCK10 and its mutually exclusive first exon isoforms were studied in human tissues and cell lines. DOCK10 is widely distributed, with prominent expression in hematopoietic tissues (spleen, thymus, leukocytes), stomach, and lungs, where both isoforms are expressed at approximately similar levels, although there are imbalances in T and B lymphocytes in favor of DOCK10.1 and DOCK10.2, respectively [9,12]. In contrast, there are striking disbalances in cell lines, which frequently show a strong preference for DOCK10.1 (e.g., HuT-78, HC-1, JY) or DOCK10.2 (e.g., 697, Jurkat, TOM-1, EHEB) [12,13]. In the present study, the expression of the alternative first exon isoforms of DOCK9 has been studied for the first time. The potential existence of such isoforms for DOCK11 has also been tested. DOCK9 and DOCK11 were widely expressed in human tissues, each with a different profile. DOCK9 was significantly expressed in tissues such as those in the lungs, placenta, uterus, thyroid, trachea, stomach, prostate, and cerebellum. Both DOCK9 isoforms were expressed at approximately similar levels in tissues, as with DOCK10. However, imbalances were found in the expression of isoforms in cell lines, since several of them only significantly expressed DOCK9.1 (e.g., Namalwa, Daudi, K-562, EHEB, DG-75, HuT-78, HeLa, REH, or MCF-7), while only one (EHEB) expressed significant levels of DOCK9.2. According to the DBCAT database, [26], the DOCK9.1 and DOCK10.1 promoters colocalize with CpG islands, suggesting that they are tightly regulated by methylation, while those of DOCK9.2 and DOCK10.2 do not, and may have looser regulation. Such alterations in expression should not be surprising, since the normal mechanisms that operate in primary tissues may be disrupted in cell lines.

DOCK11 was highly expressed in the hematopoietic tissues (leukocytes, spleen, bone marrow, and thymus), lungs and placenta. No evidence pointing to the existence of first exon isoforms of DOCK11 was found. Moreover, the good association between mRNA and protein expressions further suggests that the regulation of DOCK11 expression could be more straightforward than that of its homologs. The tissue expression profiles of the DOCK-D family members studied here and in previous studies by qRT-PCR [12,13] are comparable with global expression studies deposited in public repositories [27,28,29,30]. Combining all the data, it was found that the three genes are significantly expressed in tissues such as lung and thymus, and the main differences between the three are that DOCK9 is not expressed in the appendix or hematopoietic tissues (e.g., spleen, lymph nodes, white blood cells, or bone marrow), with the exception of T cells [8], that DOCK10 is not expressed in fat, the placenta or thyroid, and that DOCK11 is not expressed in the brain. This knowledge could be useful to identify the transcription factors targeting the DOCK9 isoforms and DOCK11—for example, through co-expression studies.

Lastly, mRNA/protein associations were tested in cell lines. The DOCK9.1 Ab and both DOCK11 Abs proved to be highly specific. However, the weaker associations between total DOCK9 mRNA and protein levels suggest that these Abs were less specific, though post-transcriptional regulation could provide an alternative explanation. In addition, the 531A Ab may be useful to study the carboxy terminal truncated isoforms of DOCK9. In summary, the Abs studied here were useful for detecting the DOCK9.1 isoform and total DOCK9 and DOCK11 proteins. Altogether, these findings represent a starting point for future studies aimed at establishing the role of DOCK9 and DOCK11 as biomarkers of disease.

## Figures and Tables

**Table 1:** Linear regression analysis between mRNA levels of DOCK9 and DOCK11 as measured by different qRT-PCR assays in human tissues.

		Tissues			Cell Lines		
Assay 1	Assay 2	R	*p* Value		R	*p* Value	
DOCK9 e1.1-e2	DOCK9 e1.2-e2	0.751	1 × 10^−5^	Significant	0.319	0.137	NS ^1^
DOCK9 e27-e28	DOCK9 e33-e34	0.913	8 × 10^−11^	Significant	0.956	1 × 10^−12^	Significant
DOCK9 e1.1-e2	DOCK9 e27-e28	0.853	3 × 10^−8^	Significant	0.708	2 × 10^−4^	Significant
DOCK9 e1.1-e2	DOCK9 e33-e34	0.757	3 × 10^−8^	Significant	0.818	2 × 10^−6^	Significant
DOCK9 e1.2-e2	DOCK9 e27-e28	0.873	6 × 10^−9^	Significant	0.824	1 × 10^−6^	Significant
DOCK9 e1.2-e2	DOCK9 e33-e34	0.687	1 × 10^−5^	Significant	0.787	8 × 10^−6^	Significant
DOCK9 e27-e28	DOCK9 e1.1-e2 + DOCK9 e1.2-e2	0.922	2 × 10^−11^	Significant	0.946	1 × 10^−11^	Significant
DOCK9 e33-e34	DOCK9 e1.1-e2 + DOCK9 e1.2-e2	0.770	4 × 10^−6^	Significant	0.987	3 × 10^−18^	Significant
DOCK11 e1-e2	DOCK11 e36-e37	0.954	4 × 10^−14^	Significant	0.970	2 × 10^−14^	Significant

^1^ NS, nonsignificant.

**Table 2:** Linear regression analysis between DOCK9 protein levels as measured by Western blot analysis using different Abs, or between protein and mRNA levels as measured by qRT-PCR using different assays, in human cell lines.

**Ab 1**	**Ab 2**	**R**	***p* Value**	
DOCK9 530A	DOCK9 531A	0.240	0.270	NS ^1^
DOCK9 530A	DOCK9 532A	0.306	0.156	NS ^1^
DOCK9 531A	DOCK9 532A	0.865	1 × 10^−7^	Significant
**Ab**	**qRT-PCR Assay**	**R**	***p* Value**	
DOCK9 530A	DOCK9 e1.1-e2	0.725	9 × 10^−5^	Significant
DOCK9 530A	DOCK9 e1.2-e2	0.054	0.808	NS ^1^
DOCK9 530A	DOCK9 e27-e28	0.310	0.151	NS ^1^
DOCK9 530A	DOCK9 e33-e34	0.504	1 × 10^−2^	Significant
DOCK9 531A	DOCK9 e1.1-e2	0.338	0.114	NS ^1^
DOCK9 531A	DOCK9 e1.2-e2	0.227	0.298	NS ^1^
DOCK9 531A	DOCK9 e27-e28	0.424	4 × 10^−2^	Significant
DOCK9 531A	DOCK9 e33-e34	0.392	6 × 10^−2^	NS ^1^
DOCK9 532A	DOCK9 e1.1-e2	0.348	0.103	NS ^1^
DOCK9 532A	DOCK9 e1.2-e2	0.121	0.583	NS ^1^
DOCK9 532A	DOCK9 e27-e28	0.329	0.126	NS ^1^
DOCK9 532A	DOCK9 e33-e34	0.346	0.106	NS ^1^

^1^ NS, nonsignificant.

**Table 3:** Linear regression analysis between DOCK11 protein levels as measured by Western blot analysis using different Abs, or between protein and mRNA levels as measured by qRT-PCR using different assays, in human cell lines.

**Ab 1**	**Ab 2**	**R**	***p* Value**	
DOCK11 638A	DOCK11 639A	0.631	1 × 10^−3^	Significant
**Ab**	**qRT-PCR Assay**	**R**	***p* Value**	
DOCK11 638A	DOCK11 e1-e2	0.646	9 × 10^−4^	Significant
DOCK11 638A	DOCK11 e36-e37	0.517	1 × 10^−2^	Significant
DOCK11 639A	DOCK11 e1-e2	0.723	1 × 10^−4^	Significant
DOCK11 639A	DOCK11 e36-e37	0.741	5 × 10^−5^	Significant

## Supplementary Materials

The following are available online at https://www.mdpi.com/2073-4468/9/3/27/s1, Table S1: Cell lines used in this work, Table S2: Plasmids used in this work, Table S3: Taqman assays used in this work, Table S4: Antibodies used in this work, Table S5: ΔCt values of different qRT-PCR assays for DOCK9 and DOCK11 in human tissues and cell lines.
