# Supplementary material for: Expression of DOCK9 and DOCK11 Analyzed with Commercial Antibodies: Focus on Regulation of Mutually Exclusive First Exon Isoforms
Source: Antibodies (Basel). 2020 Jun 27;9(3):27. doi: 10.3390/antib9030027 (PMC7551865; doi:10.3390/antib9030027)
Supplement: Supplementary file 1 [file antibodies-09-00027-s001.pdf]

Supplementary Information:

Table S1. Cell lines used in this work

Cell line	Cell lineage	Cell origin	Source*
Jurkat	T lymphocytes	Acute lymphoblastic leukemia	1
HuT-78	T lymphocytes	Sezary syndrome	2
Mec-1	B lymphocytes	Chronic lymphocytic leukemia	3
EHEB	B lymphocytes	Chronic lymphocytic leukemia	3
Bonna-12	B lymphocytes	Hairy cell leukemia	4
HC-1	B lymphocytes	Hairy cell leukemia	4
697	B lymphocytes	Acute lymphoblastic leukemia	5
RS4;11	B lymphocytes	Acute lymphoblastic leukemia	5
REH	B lymphocytes	Acute lymphoblastic leukemia	5
TOM-1	B lymphocytes	Acute lymphoblastic leukemia	5
Namalwa	B lymphocytes	Burkitt lymphoma	6
DG-75	B lymphocytes	Burkitt lymphoma	7
Daudi	B lymphocytes	Burkitt lymphoma	1
PER	B lymphocytes	Epstein-Barr virus-transformed lymphoblasts	6
JY	B lymphocytes	Epstein-Barr virus-transformed lymphoblasts	8
NB-4	Myeloid	Acute myeloid leukemia	1
HL-60	Myeloid	Acute myeloid leukemia	1
Kasumi-1	Myeloid	Acute myeloid leukemia	1
K-562	Myeloid	Chronic myeloid leukemia	1
P39	Myeloid	Myelodysplastic syndrome	5
Hep-G2	Epithelial	Hepatocellular carcinoma	1
MCF-7	Epithelial	Breast adenocarcinoma	9
HeLa	Epithelial	Cervix carcinoma	10
293T	Uncertain (fibroblastic, epithelial, neuronal…)	Embryonic kidney	1

1. Given by Dr. Christine Chomienne, Saint-Louis Hospital, Paris, France.
2. Given by Dr. José Zamorano, San Pedro de Alcántara Hospital, Cáceres, Spain.
3. Given by Dr. Rose Ann Padua, Saint-Louis Hospital, Paris, France.
4. Given by Dr. Francesc Bosch, Clinic Hospital, Barcelona, Spain.
5. Purchased from DSMZ-German Collection of Microorganisms and Cell Cultures.
6. Given by Dr. Maryline Sasportes, Saint-Louis Hospital, Paris, France.
7. Given by Dr. Berthold Henglein, Institut Curie, Paris, France.
8. Given by Dr. José Antonio Campillo, Immunology Service, Clinic University Hospital Virgen de la Arrixaca, Murcia, Spain.
9. Given by Dr. Juan Cabezas, IMIB-Arrixaca, Murcia, Spain.
10. Given by Dr. José Yélamos, Experimental Surgery Unit, Clinic University Hospital Virgen de la Arrixaca, Murcia, Spain.

Table S2. Plasmids used in this work

Vector	NCBI acc. no.	Reference
pEF-FLAG-DOCK9	NM_015296.2	Meller et al., 2004
pSG5-DOCK10.1	NM_014689	Ruiz-Lafuente et al., 2015
pSG5-DOCK11	NM_144658.3	Ruiz-Lafuente et al., 2015

Table S3. Taqman assays used in this work

Gene, isoform	Applied Biosciences Reference	RefSeq ID	Exons	Function	Sequence
DOCK9	Hs_00324508_m1	NM_015296	27-28	Probe (FAM)	5'- AGTTAAGTTGCTGCGAAACCAGAGA -3'
DOCK9	Hs_01004254_m1	NM_015296	33-34	Probe (FAM)	5'- CCATCTCCGGCATTGCTTCTCCATA -3'
DOCK9.1	Hs_01004241_m1	NM_015296	1.1-2	Probe (FAM)	5'- GGCTCCGTGCTCCTGGCAAAGCCAA -3'
DOCK9.2	Hs_00392677_m1	NM_001130048	1.2-2	Probe (FAM)	5'- CCTGTGCCGGCAAAGCCAAAGCTAA -3'
DOCK11	Custom	NM_144658	1	PCR, forward	5'- CCGGCAGAGCGTGTCT -3'
			2	PCR, reverse	5'- TGGGCAATAACATTCTCATAGTCCA -3'
			1-2	Probe (FAM)	5'- TTGGCCTTTTCCAGCACCAC -3'
DOCK11	Hs00376176_m1	NM_144658	36-37	Probe (FAM)	5'- CTTATACTTTTAGAAGTATGCTTGT -3'
GAPDH	Hs99999905_m1	NM_002046	3	Probe (FAM)	5'- GGGCGCCTGGTCACCAGGGCTGCTT -3'

Table S4. Antibodies used in this work

Protein target	Amino acid positions	Dilution (ratio)	Source	Catalog No.	Manufacturer
DOCK9	1-50	1:2000	Rabbit	A300-530A	Bethyl Laboratories
DOCK9	1250-1300	1:2000	Rabbit	A300-531A	Bethyl Laboratories
DOCK9	1850-1900	1:2000	Rabbit	A300-532A	Bethyl Laboratories
DOCK10	100-150	1:5000	Rabbit	A301-305A	Bethyl Laboratories
DOCK11	100-150	1:2000	Rabbit	A301-638A	Bethyl Laboratories
DOCK11	400-450	1:5000	Rabbit	A301-639A	Bethyl Laboratories
GAPDH (FL-335, HRP)		1:1000	Rabbit	sc-25778	Santa Cruz Biotechnology
Rabbit Igs (HRP)		1:2000	Swine	P0399	Dako

Table S5. ΔCt values of different QRTPCR assays for DOCK9 and DOCK11 in human tissues and cell lines

Assay	Tissues					Cell lines				
	Average	Range				Average	Range			
		High	Tissue	Low	Tissue		High	Cell line	Low	Cell line
DOCK9 e1.1-e2	6.55	4.16	Lung	12.39	Sk. muscle	12.48	7.64	Namalwa	23.28	HL-60
DOCK9 e1.2-e2	8.04	4.36	Lung	16.36	Leukocytes	18.58	6.86	EHEB	23.28	HL-60
DOCK9 e27-e28	5.70	3.09	Lung	10.54	Sk. muscle	10.26	4.74	EHEB	21.65	P39
DOCK9 e33-e34	5.07	2.89	Lung	10.05	Sk. muscle	10.49	5.04	EHEB	21.97	P39
DOCK11 e1-e2	6.32	2.85	Leukocytes	10.86	Sk. muscle	7.93	5.24	TOM-1	13.91	Hep-G2
DOCK11 e36-e37	8.88	5.90	Leukocytes	13.67	Sk. muscle	8.29	5.86	RS4;11	14.45	Hep-G2
